# Rescuing missing data in connectome-based predictive modeling

**DOI:** 10.1162/imag_a_00071

**Published:** 2024-02-02

**Authors:** Qinghao Liang, Rongtao Jiang, Brendan D. Adkinson, Matthew Rosenblatt, Saloni Mehta, Maya L. Foster, Siyuan Dong, Chenyu You, Sahand Negahban, Harrison H. Zhou, Joseph Chang, Dustin Scheinost

**Affiliations:** Department of Biomedical Engineering, Yale University, New Haven, CT, United States; Department of Radiology & Biomedical Imaging, Yale School of Medicine, New Haven, CT, United States; Interdepartmental Neuroscience Program, Yale University, New Haven, CT, United States; Department of Electrical Engineering, Yale University, New Haven, CT, United States; Department of Statistics & Data Science, Yale University, New Haven, CT, United States; Child Study Center, Yale School of Medicine, New Haven, CT, United States

**Keywords:** Functional connectivity, missing data, imputation, machine learning, fMRI

## Abstract

Recent evidence suggests brain-phenotype predictions may require very large sample sizes. However, as the sample size increases, missing data also increase. Conventional methods, like complete-case analysis, discard useful information and shrink the sample size. To address the missing data problem, we investigated rescuing these missing data through imputation. Imputation is substituting estimated values for missing data to be used in downstream analyses. We integrated imputation methods into the Connectome-based Predictive Modeling (CPM) framework. Utilizing four open-source datasets—the Human Connectome Project, the Philadelphia Neurodevelopmental Cohort, the UCLA Consortium for Neuropsychiatric Phenomics, and the Healthy Brain Network (HBN)—we validated and compared our framework with different imputation methods against complete-case analysis for both missing connectomes and missing phenotypic measures scenarios. Imputing connectomes exhibited superior prediction performance on real and simulated missing data compared to complete-case analysis. In addition, we found that imputation accuracy was a good indicator for choosing an imputation method for missing phenotypic measures but not informative for missing connectomes. In a real-world example predicting cognition using the HBN, we rescued 628 individuals through imputation, doubling the complete case sample size and increasing the variance explained by the predicted value by 45%. In conclusion, our study is a benchmark for state-of-the-art imputation techniques when dealing with missing connectome and phenotypic data in predictive modeling scenarios. Our results suggest that improving prediction performance can be achieved by strategically addressing missing data through effective imputation methods rather than resorting to the outright exclusion of participants. Our results suggest that rescuing data with imputation, instead of discarding participants with missing information, improves prediction performance.

## 1 Introduction

Reproducible brain-wide associations require sample sizes on the order of thousands of individuals (Genon et al., 2022; Liu et al., 2023; Marek et al., 2022) due to the small effect sizes of brain-phenotype predictions. Using data from multiple sources increases the effect sizes of brain-phenotype predictions (Dubois et al., 2018; Elliott et al., 2019; Gao et al., 2019; Jiang et al., 2020; Mellem et al., 2020; Ooi et al., 2022; Yu et al., 2020). For example, supervised learning models combining different multiple task connectomes outperform those built from a single connectome (Barron et al., 2021; Gao et al., 2019; Garrison et al., 2023), and latent variables derived from a battery of phenotypic measures are more predictable as compared to a single phenotypic measure (Barron et al., 2021; Dubois et al., 2018; Garrison et al., 2023; Rapuano et al., 2020). These results suggest that using connectomes and behavioral data from multiple sources is a powerful approach to characterizing brain-phenotype relationships.

Nevertheless, there is a significant tradeoff when using multiple data sources—the amount of missing data increases. Currently, most fMRI studies only consider complete-case data, meaning that participants with any missing phenotypic or imaging data are removed from the analysis. Thus, there is a substantial decrease in sample size. Retaining more participants in studies is needed to improve the statistical power (Genon et al., 2022; Marek et al., 2022). Further, for infants, the elderly, and those with psychiatric disorders, acquiring neuroimaging data is especially difficult and costly (Korom et al., 2022; Tejavibulya et al., 2022). Discarding participants with missing data wastes resources.

Despite being widespread in many domains of biomedicine, handling missing data is nascent in supervised learning. Most extant supervised learning studies inadequately report or handle missing data (Nijman et al., 2022). In addition, most of the literature on missing data focuses on statistical inference instead of supervised learning (Perez-Lebel et al., 2022). Only a handful of studies touch upon the theories (Josse et al., 2020) or evaluate supervised learning with missing values (Batista & Monard, 2003; Perez-Lebel et al., 2022; Poulos & Valle, 2018; Tresp et al., 1994; Zhang et al., 2010). However, the systematic evaluation of supervised learning with missing values in predictive modeling of functional connectivity data is crucial to maximizing the utility of expensive neuroimaging datasets even in the presence of missing data.

Data imputation, or substituting missing data with estimated values for downstream analyses, is a standard way to rescue missing data. Three types of missingness mechanisms exist—missing completely at random (MCAR), missing at random (MAR), and missing not at random (MNAR) (Little & Rubin, 2019). Due to the specialties of connectome data, many popular imputation methods—including statistical models like multiple imputation, maximum likelihood estimation, and fully Bayesian methods (Baraldi & Enders, 2010)—are not suitable. In connectome-based prediction, the input features (edges in connectomes) are usually in a high dimension (~30 k), but the sample size is small. Even after feature selection, the dimension is still high relative to the sample size (Rosenberg et al., 2018). The connectome features are highly correlated and noisy and encode phenotypic information across many edges (Jiang, Scheinost, et al., 2022; Jiang, Woo, et al., 2022). Moreover, the features are missed in a block-wise pattern since a whole connectome is missed. However, the redundancy in different task connectomes can be leveraged by simple imputation methods with low computational costs. Unlike connectome data imputation, most state-of-the-art imputation methods are suitable for the case of missing phenotypic measures.

This work incorporated imputation methods into connectome-based predictive modeling (CPM) to rescue missing data in functional connectomes and phenotypic measures. We investigated the performance of several imputation strategies in four large open-source datasets with multiple fMRI tasks—the Human Connectome Project (HCP) (Van Essen et al., 2012), the Philadelphia Neurodevelopmental Cohort (PNC) (Poldrack et al., 2016), the UCLA Consortium for Neuropsychiatric Phenomics (CNP) (Satterthwaite et al., 2016), and the Healthy Brain Network (HBN) (Alexander et al., 2017). All four datasets contain a subset of participants with missing fMRI data in specific tasks due to the unavailability or quality of the scans. Additionally, the HBN dataset includes a substantial missing data rate due to the difficulty in scanning children with mental health conditions. We assessed the effectiveness of imputation methods in handling missing connectome data when predicting fluid intelligence, age, and sex.

Specifically, we performed the following experiments to investigate the impact of rescuing missing data in functional connectomes and phenotypic measures for predictive modeling. Using the HCP, PNC, and CNP datasets, we evaluated the improved performance of models built on datasets with missing connectome data compared to the complete case. Using the HBN dataset, the only dataset with significant missing phenotypic data, we investigated if imputing missing phenotypic data improves prediction performance. Using the HCP dataset, we simulated the impact of missingness rates and mechanism for missing connectomes and phenotypic data. Finally, using the HBM, we provided a real-world example of the application of our approach and imputed both connectome and phenotypic data in the HBN. When used appropriately, data imputation is a valuable addition to neuroimaging-based prediction pipelines.

## 2 Materials and Methods

### 2.1 Predictive modeling using data with missing values

**Fig. 1.**
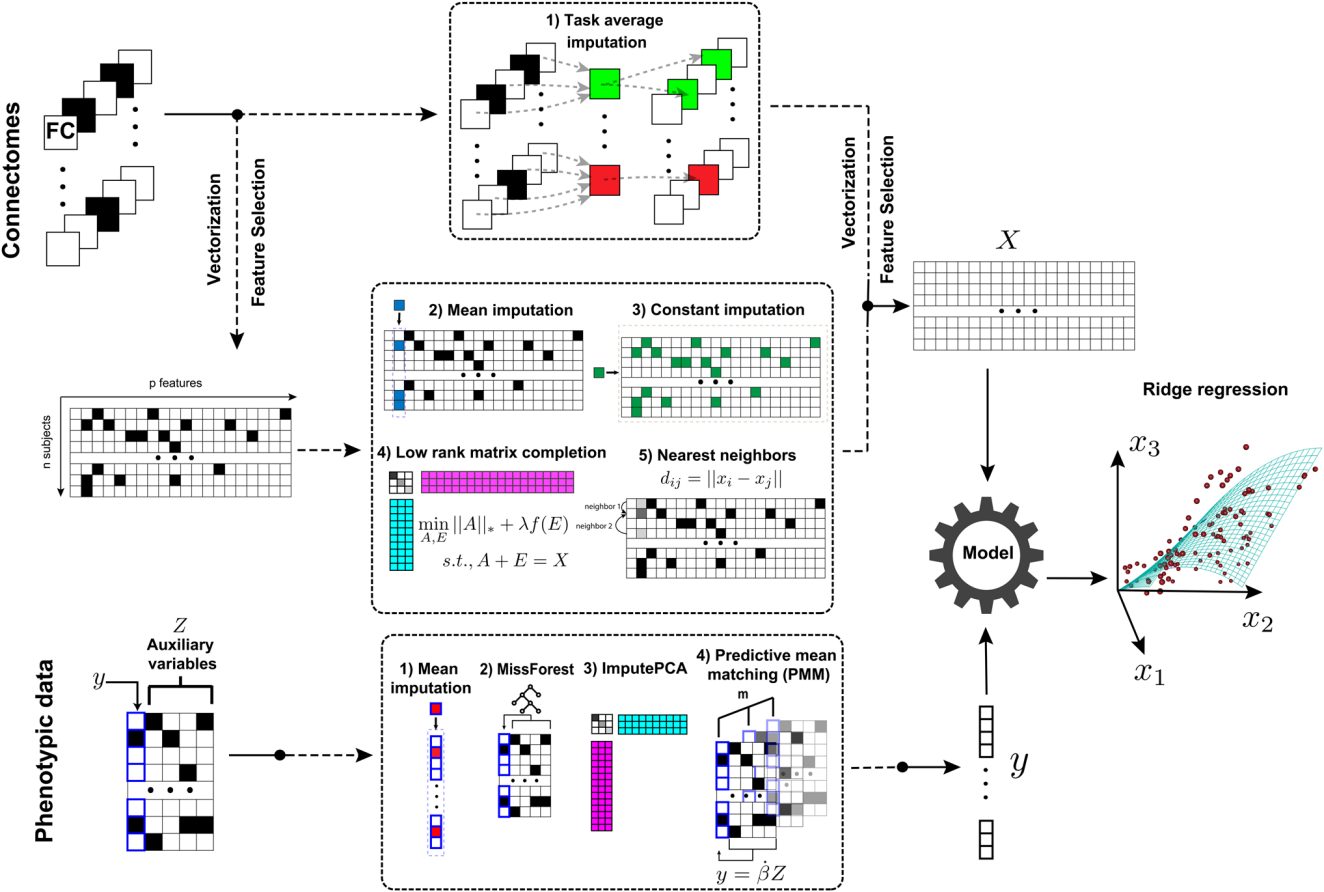
Predictive modeling framework for handling missing data. Missing connectomes are rescued using 1) Task average replacement, 2) Mean imputation, 3) Constant value imputation, 4) Robust Matrix Completion, or 5) Nearest Neighbors imputation. Missing phenotypic measures are imputed with auxiliary variables using 1) Predictive mean matching (PMM), 2) ImputePCA, 3) MissForest, or 4) Mean imputation. The phenotypic and connectivity data are then used in standardized predictive models such as ridge regression or support vector machine. The black squares represent missing data.

We modified Connectome-based Predictive Modeling (CPM) to rescue data with missing connectomes by incorporating imputation methods to fill in the missing values before models were trained on the completed data. In CPM, the initial step involves vectorizing the connectomes of each subject and concatenating them across all tasks to create a single input vector. Subsequently, significant features for prediction are selected using complete data through univariate methods. For regression, we calculated the Pearson correlation between each feature and the phenotype of interest, while for classification, we calculated the F-value for ANOVA comparing each feature and the phenotype of interest. Features with significant associations are selected for predictive modeling. Figure 1 illustrates the missing data handling process in predictive modeling.

#### 2.1.1 Missing connectome data (missing X data)

Five imputation methods were used to fill in missing values. All other methods except for task average imputation were applied to selected features to reduce the computational burden. The imputation methods are detailed below:

(1) Task average imputation: For subject i, the mean of all observed task connectomes was calculated as $C_{i}$. Then, all missing selected features of subject i were replaced with the corresponding value of $C_{i}$.
(2) Mean imputation: Each missing value was replaced by the mean of the observed values along its column. Mean imputation was implemented using the python package scikit-learn (Pedregosa et al., 2011).
(3) Constant values imputation: All missing values were imputed with a constant value. Here, we chose the constant value as the mean of all observed entries. Constant value imputation was implemented using the Python package scikit-learn (Pedregosa et al., 2011).
(4) Robust matrix completion: Given the high collinearity of edges in functional connectomes, we assume the predictive information is encoded into a lower dimensional space. Robust matrix completion recovers the low-rank matrix when part of the entries is observed and corrupted by noise (Shang et al., 2014). Robust matrix completion could be formulated as the following convex optimization problem:
  $$min_{A,E}\;||\;A\;|{|}_{*}+\lambda f\left(E\right),s.t.,A+E=Xss$$
  where X is the corrupted input matrix (the selected features in our case), $||\;A\;|{|}_{*}$ is the trace norm of the desired matrix A (output), λ is the parameter that controls the noise level, E is the noise matrix, and f(⋅) denotes the loss function. We chose the $l_{2}$-norm loss in this study because the noises spread across many entries of X. The implementation details of this algorithm can be found in Liang et al. (2021).
(5) Nearest neighbors imputation: Nearest neighbors imputation was performed by computing the Euclidean distance metric that supports missing values to find the nearest neighbors for each subject. For each missing value, values from the k nearest neighbors with a value for the same feature were used to impute the missing value. The neighbors' values were either averaged uniformly or weighted by the distance to each neighbor. If a subject had more than one missing feature, then the neighbors for that subject could be different depending on the feature being imputed. We chose k=5 and used a uniform weight for each neighbor in this study. The nearest-neighbors imputation was implemented using the Python package scikit-learn (Pedregosa et al., 2011).

#### 2.1.2 Missing phenotypic data (missing y data)

When a single phenotypic measure y was to be predicted, we utilized a set of auxiliary variables Z=$\left(z_{1},z_{2},\ldots ,z_{k}\right)$ correlated with y that could also contain missing data to impute *y*. When the first principal component of the variables of interest $Y=\left(y_{1},y_{2},\ldots ,y_{k}\right)$ was to be predicted, we imputed all missing values in Y and applied principal component analysis (PCA) to obtain the first principal component. The PCA coefficients were then applied to the testing set. The imputation methods are detailed below:

(1) Mean imputation: Each missing value was replaced by the mean of the observed values along its column.
(2) Random forest algorithm for missing data imputation (MissForest): MissForest is a non-parametric imputation method for mixed-type data. For each variable in the dataset, MissForest fits a random forest on the observed part and then predicts the missing part (the predicted values were later used in training models of other variables). The algorithm repeats these two steps until a stopping criterion (accuracy of fitting the observed part) is met. The algorithm was implemented in the R package “missForest.”
(3) Regularized iterative principal component analysis (ImputePCA): Iterative Principal Component Analysis (PCA) algorithm, also known as the Expectation-maximization PCA (EMPCA) algorithm, is an expectation-maximization algorithm for a PCA fixed-effects model, where data are generated as a fixed structure having a low-rank representation corrupted by noise (Josse & Husson, 2016). Regularized iterative Principal Component Analysis uses regularized methods to tackle the overfitting problems when data are noisy and there are many missing values. The algorithm was implemented in the function imputePCA in the R package “missMDA.”
(4) Predictive mean matching (PMM): Multiple imputation by chained equations (White et al., 2011) is a robust, informative method of dealing with missing data in datasets. The procedure imputes missing data in a dataset through an iterative series of predictive models. In each iteration, each specified variable in the dataset is imputed using the other variables. These iterations are run until convergence has been achieved. PMM selects non-missing samples with predictive values close to the predictive value of the missing sample. The closest *N* values are chosen as candidates, from which a value is chosen randomly. *M* imputation values are generated using different random initializations, and the mean value is taken to fill in the missing entries for predictive modeling. The algorithm used the R package “mice” (Buuren & Groothuis-Oudshoorn, 2011).

#### 2.1.3 Validation methods

Models were evaluated using 10-fold cross-validation. Complete-case analysis served as the baseline. Participants with missing data (X or y) were included only in the training set. The testing set consisted only of participants with complete data. Additionally, we assessed models on participants with missing X by randomly splitting all participants into training and testing sets.

Notably, missing data from the training and testing sets were imputed independently to prevent data leakage. A feature selection threshold of p < 0.01 was used to select features in the training set. The ridge regression hyper-parameter was determined by grid searching using nested 5-fold cross-validation within the training set. For regression, the model performance was evaluated by the cross-validated $R^{2},\;\;R_{\;\;\;\;CV}^{2}=1-\sum_{i=1}^{n}{\left(y_{i}-\hat{y}\right)}^{2}/\sum_{i=1}^{n}{\left(y_{i}-\bar{y}\right)}^{2}.$ can be negative, which suggests the predictive model performs worse than simply guessing the mean of the phenotypic measure. In this case, we set $R_{\;\;\;\;CV}^{2}$ to 0 (Scheinost et al., 2019; Sui et al., 2020). For classification, the model performance was evaluated by each fold's average Area Under the Receiver Operating Characteristic Curve (ROC AUC).

### 2.2 Datasets

Four datasets were used in our study: the Human Connectome Project (HCP) 900 Subject Release, the UCLA Consortium for Neuropsychiatric Phenomics (CNP), the Philadelphia Neurodevelopmental Cohort (PNC), and the Healthy Brain Network (HBN). The HCP dataset was used for the analysis of real missing connectomes and the simulations of missing connectomes and phenotypic measures. The CNP and PNC were used for only the analysis of real missing connectomes. The HBN dataset was used for the analysis of real missing connectomes and phenotypic measures. We chose fluid intelligence as the primary variable to predict based on several key factors. Firstly, fluid intelligence is a robust measure of general cognitive ability. Secondly, it is commonly collected in most datasets and frequently employed as a benchmark in various studies to evaluate and compare modeling methods. Lastly, predicting fluid intelligence allows us to uncover the neurological foundations of individual cognitive differences, enhancing our understanding of human cognition.

**HBN participants.** The HBN dataset incorporated high-resolution fMRI data in 2 distinct task conditions and a battery of cognitive measures. Participants performed two naturalistic viewing sessions of movie clips from “Despicable Me” and “The Present” in the scanner. Ten cognitive measures were used for prediction: the Flanker Inhibitory Control and Attention (Flanker), List Sorting Working Memory (ListSort), Pattern Comparison Processing Speed (ProcSpeed), and Card Sort tests (CardSort) were selected from the NIH Toolbox; the Math Problem Solving and Numerical Operations subtests were selected from the Wechsler Individual Achievement Test (WIAT); and the Fluid Reasoning, Visual Spatial Reasoning, Processing Speed, and Working Memory subtests were selected from the Wechsler Intelligence Scale for Children (WISC-V) (Alexander et al., 2017).

**HCP participants.** We used the released HCP S900 data, which incorporated high-resolution fMRI data in 7 distinct task conditions (gambling, language, motor, relational, social, working memory, and emotion), and a battery of cognitive tests. The matrix reasoning test (PMAT)—a measure of fluid intelligence—was used as the phenotypic measure for prediction. For the simulations of missing phenotypic measures, we used the unadjusted score of 10 cognitive measures from Dubois et al. (2018) (PicVocab, PMAT, ReadEng, VSPLOT, IWRD, PicSeq, ListSort, Flanker, CardSort, and ProcSpeed).

**CNP participants.** The CNP dataset incorporated high-resolution fMRI data in 6 distinct task conditions (balloon analog risk task, paired-associative memory encoding, paired-associative memory retrieval, spatial working memory capacity, stop signal, and task switching), and the fluid intelligence measure. Fluid intelligence was assessed by WAIS-IV matrix reasoning (Poldrack et al., 2016).

**PNC participants.** We used the first released PNC data, which incorporated high-resolution fMRI data in 2 distinct task conditions (working memory and emotion), and the fluid intelligence measure. Fluid intelligence was assessed by the 24-item version of the Penn Matrix Reasoning Test (Moore et al., 2015).

#### 2.2.1 fMRI processing

We followed an established pipeline to create connectome data (Barron et al., 2021). Functional images were motion-corrected using SPM12. All further analyses were completed using BioImage Suites. Several covariates of no interest were regressed from the data, including linear and quadratic drifts, mean cerebral-spinal-fluid (CSF) signal, mean white-matter signal, and mean gray matter signal. For additional control of possible motion-related confounds, a 24-parameter motion model (including six rigid-body motion parameters, six temporal derivatives, and these terms squared) was regressed from the data. The data were temporally smoothed with a Gaussian filter (approximate cutoff frequency = 0.12 Hz).

The brain was parcellated into 268 macroscale regions of interest using a whole-brain, functional atlas defined in a separate sample (Shen et al., 2013). For each subject, the regional time series were calculated by averaging the voxel-wise fMRI time series in a node. The pairwise Pearson correlation between all node time series was calculated and Fisher z-transformed, yielding a 268 by 268 matrix for each scan.

#### 2.2.2 Missing data

For all datasets, connectomes were classified as missing if the imaging data were unavailable (such as subject dropouts or unshared data) or failed quality control (such as high motion or missing brain sections). Scans with a frame-to-frame displacement greater than 0.15 mm were deemed to have high motion. For analyses that included real missing data, we retained participants who had at least one connectome, along with measures of age, sex, and fluid intelligence. For analyses with simulated missing connectomes, we used the complete-case data from the HCP dataset. Details of the HCP, CNP, and PNC datasets used in the experiments are summarized in Table 1, while the missing information for each specific task in each dataset is summarized in Figure S1 of the Supplementary Material.

**Table 1.** Summary of datasets (HCP, PNC, CNP) used in missing connectome data analysis.

		N	Sex (male:female)	Age range (years)
HCP	Complete-case	645	296:349	22-37
	All data	855	379:476	22-37
CNP	Complete-case	152	85:67	21-50
	All data	244	139:105	21-50
PNC	Complete-case	682	310:372	8-21
	All data	813	373:440	8-21

For analyses involving missing phenotypic data, we retained participants who had at least one observed phenotypic variable of interest. For analyses with real missing phenotypic data, we used participants with at least one cognitive measure from the HBN dataset. For analyses with simulated missing phenotypic data, we used participants with complete connectome data and all 10 cognitive measures from the HCP dataset. Details of the HBN and HCP datasets used in these experiments are summarized in Table 2.

**Table 2.** Summary of datasets (HBN, HCP) measures used in missing phenotypic data analysis.

		N	Sex (male:female)	Age range (years)
HBN	Complete-case	698	447:251	5-21
	Complete connectome data	893	575:318	5-22
	All data	1326	851:475	5-22
HCP	Complete-case	514	240:274	22-37

### 2.3 Experiments

#### 2.3.1 Analyses with real missing connectomes

We utilized our pipeline to analyze the HCP, CNP, and PNC datasets, and performed 100 iterations of 10-fold cross-validation to predict fluid intelligence, age, and sex. The performance of each imputation method, as well as a complete-case analysis, was calculated and reported.

#### 2.3.2 Analyses with simulated missing connectomes

We conducted simulation tests to investigate the impact of different missingness rates and missing data mechanisms on the performance of imputation strategies. For our experiment, we simulated two missing data scenarios. In the first scenario, we randomly deleted connectomes (MCAR). In the second scenario, we deleted connectomes with a probability proportional to the motion of the original scan, making the data missing not at random (MNAR) as the missingness was related to the data itself. We tested missingness rates ranging from 0% to 80%, averaged across the entire dataset. We performed the simulation study on the complete-case of the HCP dataset, as it has the largest amount of complete-case data. For each missingness rate, we generated 500 missingness patterns using different random seeds. For each missingness pattern, we conducted 10-fold cross-validation with a random data partition to evaluate the performance of CPM, and since the ground truth of the missing values, $x_{true}$, was known, we also calculated the imputation accuracy of each imputation method. We assessed the accuracy of the different imputation methods using the normalized-root-mean-square error (NRMSE) between the ground truth and the imputed values $x_{impute}$. $NRMSE=\frac{RMSE}{\sigma }$, where the root-mean-square error (RMSE) is calculated as $\sqrt{\frac{{\left(x_{true}-x_{impute}\right)}^{2}}{N}}$ (N is the number of entries in $x_{true}$) and σ is the standard deviation of $x_{true}$.

#### 2.3.3 Analyses with real missing phenotypic data

We applied our pipeline to the HBN datasets with 100 iterations of 10-fold cross-validation. Participants with complete connectome data and missing executive function were used for predicting Flanker, ProcSpeed, CardSort, and Working Memory. For predicting a latent factor of cognition, we examined the performance of the models imputing cognitive measures and models imputing both cognitive measures and connectomes. Model performance of each imputation method and a complete-case study were calculated and reported.

#### 2.3.4 Analyses with simulated missing phenotypic data

We generated 100 missingness patterns in the 10 cognitive measures of the complete-case HCP data by deleting values completely at random (MCAR). Missingness rates ranged from 0% to 40%. Fluid intelligence was used as the target variable to predict, while the other nine were used as auxiliary variables. Ten iterations of CPM were run for each missingness pattern to calculate the model performance and imputation accuracy as described above.

## 3 Results

### 3.1 Imputing missing connectomes improves prediction performance

**Fig. 2.**
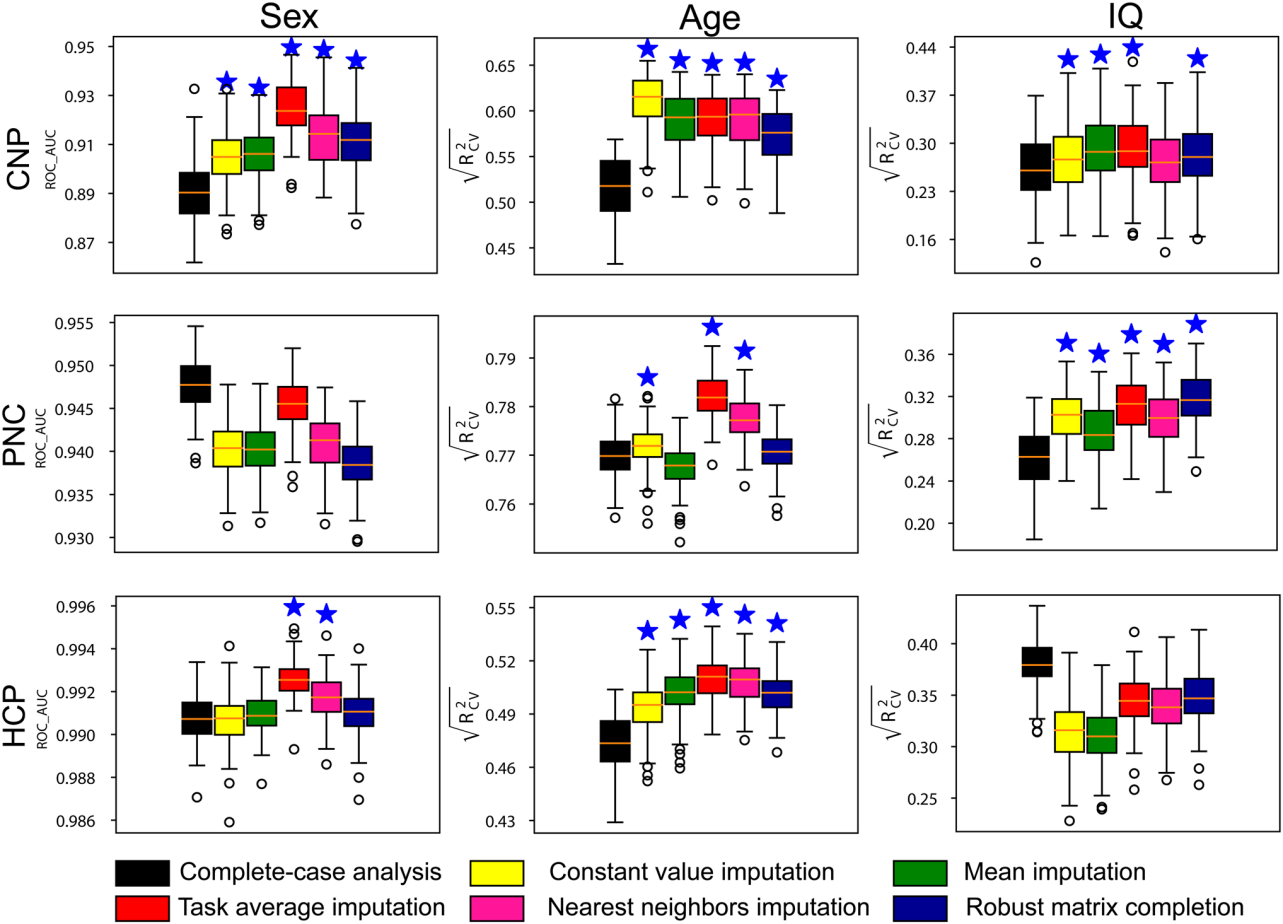
Prediction performance of models built on datasets with missing connectome data. The testing set only includes participants with complete data. Prediction performance of sex, age, and fluid intelligence based on data imputed using multiple imputation strategies in three datasets, including CNP, PNC, and HCP. Stars above the boxplots indicate a significantly higher prediction performance relative to complete-case analysis (p < 0.001).

Using real missing data, we investigated if imputing missing connectomes improves prediction performance. Figure 2 presents the prediction performance of phenotypic measures (sex, age, IQ) based on complete-case data and data imputed using multiple methods. Our results demonstrate that imputing missing data can significantly improve prediction accuracy compared to models built on complete data, in most cases. For sex classification, all models achieved high accuracy (>0.9) across all three datasets. Imputation improved model performance most significantly on CNP, where complete-case analysis had relatively low accuracy (0.900 ± 0.008). However, for PNC and HCP datasets, where complete-case analysis already achieved very high accuracy, data imputation only had a marginal effect on prediction. For age and fluid intelligence (IQ) prediction, imputation led to improved performance in most cases, except for the HCP dataset. Task average imputation was found to achieve the best performance among all imputation methods. However, the relative performance of different imputation methods varies across datasets and prediction tasks. Additionally, the impact of the feature selection threshold on different models is shown in Figure S3. Results indicated that after selecting enough features, the performance of models was relatively stable across different methods. Notably, the results presented here were derived from models where only participants with complete data were included in the testing set. Our sensitivity analysis including participants with incomplete data yields similar findings (Fig. S2). In some cases, the performance decline in comparison to complete-case analysis may be attributed to the disproportionate missing of critical information in the participants added to the training set (Fig. S4).

### 3.2 Simulation to investigate the impact of missingness rates and mechanisms

**Fig. 3.**
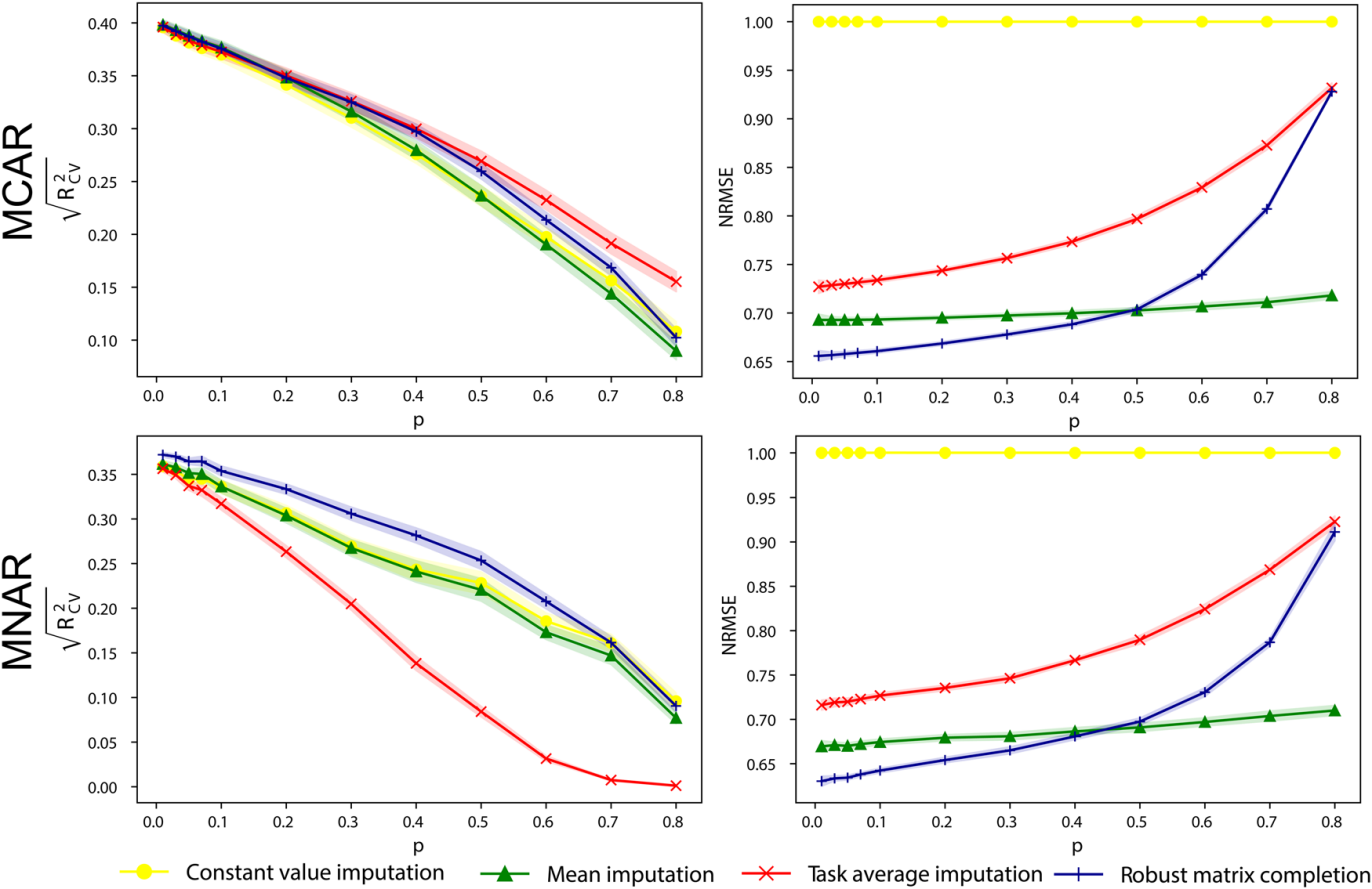
Prediction performance of models built with participants of simulated missing connectomes. Prediction performance $\left(\sqrt{R_{CV}^{2}}\right)$ of models predicting IQ using different missing data handling strategies as a function of missingness rates *p*. Imputation accuracy (NRMSE) as a function of missingness rates *p*. The shadow areas were 95% confidence intervals. The missing data were generated on the complete data in HCP through random sampling (MCAR) or sampling.

We investigated the impact of missingness rates and mechanisms on imputation accuracy and prediction performance using the HCP complete-case data. As shown in Figure 3, all imputation strategies demonstrate impaired prediction of IQ as missingness rates increase. For MCAR, task average imputation outperformed other methods at high missingness rates, while all methods had a similar performance at low rates. For MNAR, robust matrix completion had the highest performance, while task average imputation performed the worst. There was no significant difference in terms of imputation accuracy between the two missing mechanisms. The accuracy of imputation methods in recovering connectome data decreased with increasing missingness rates. Notably, task average imputation showed the worst performance in recovering connectome data. Robust matrix completion was the best method for recovering connectome data for missingness rates below 0.4. However, it showed impaired performance for missingness rates above 0.4.

### 3.3 Imputing missing phenotypic data improves prediction performance

**Fig. 4.**
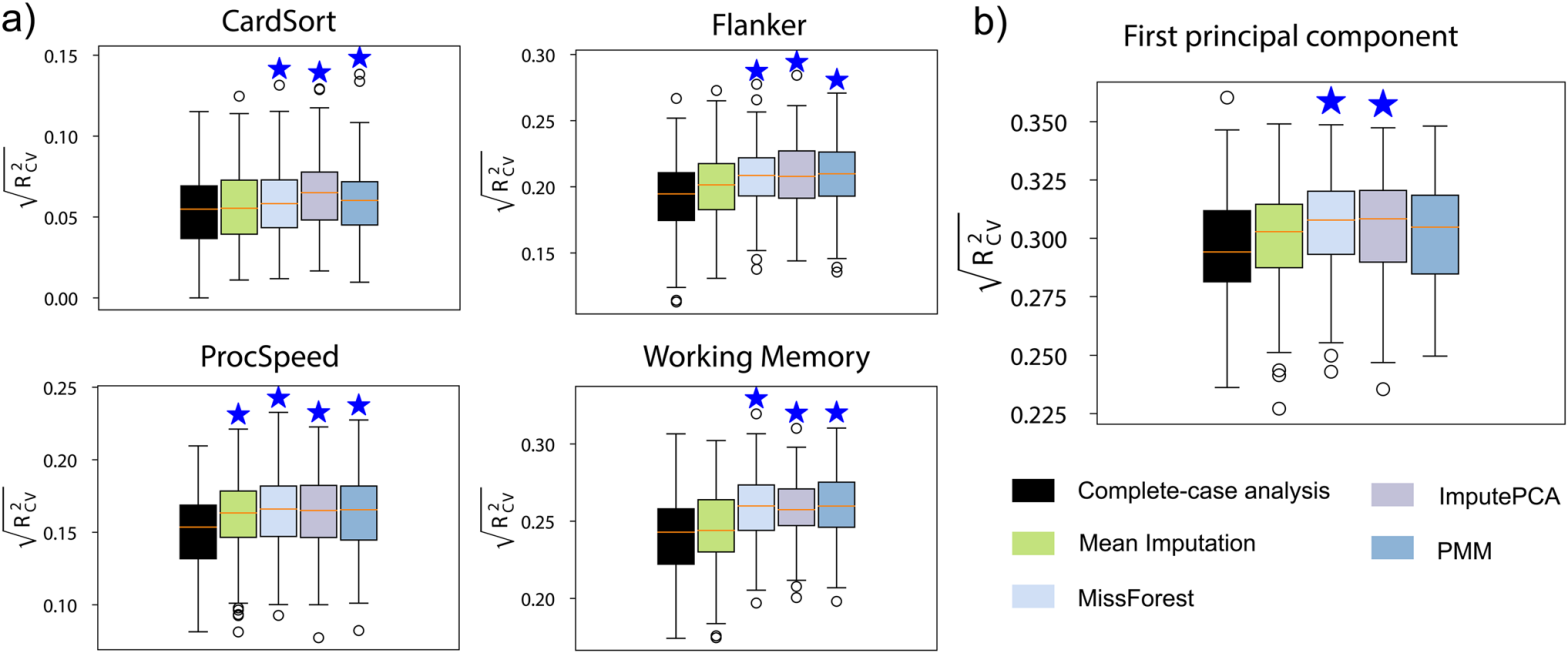
Prediction performance of models built with HBN participants with missing phenotypic measures. (a) Performance of predicting a single cognitive measure. (b) Performance of predicting the first principal component of 10 cognitive measures.

In the HBN dataset, a substantial number of participants have missing connectomes and cognitive measures. Through imputation, we were able to rescue several participants: 33 for CardSort, 34 for Flanker, 42 for ProcSpeed, and 54 for Working Memory. For predicting the first principal component of 10 cognitive measures, 195 participants were rescued by imputing the missing cognitive measures. Only participants with missing data were included in the training set, and the prediction performance was evaluated on the same testing set used for complete-case analysis. Given the utilization of two multiple imputation techniques (imputePCA and PMM), we explored the feasibility of consolidating the performance of models constructed on each imputed dataset, rather than relying solely on the averaged imputed dataset. As depicted in Figure S5, integrating the mean of multiple imputed datasets for predictive modeling yielded more robust outcomes than compiling the performance across all imputed datasets. This averaging approach reduced the inherent imputation uncertainty and augmented the precision of estimating missing data. As depicted in Figure 4a and b, imputing missing cognitive measures significantly improved the prediction performance compared to complete-case analysis. Among the four imputation methods used, mean imputation yielded the lowest prediction performance.

### 3.4 Simulation to investigate the impact of missingness rates in phenotypic measures

**Fig. 5.**
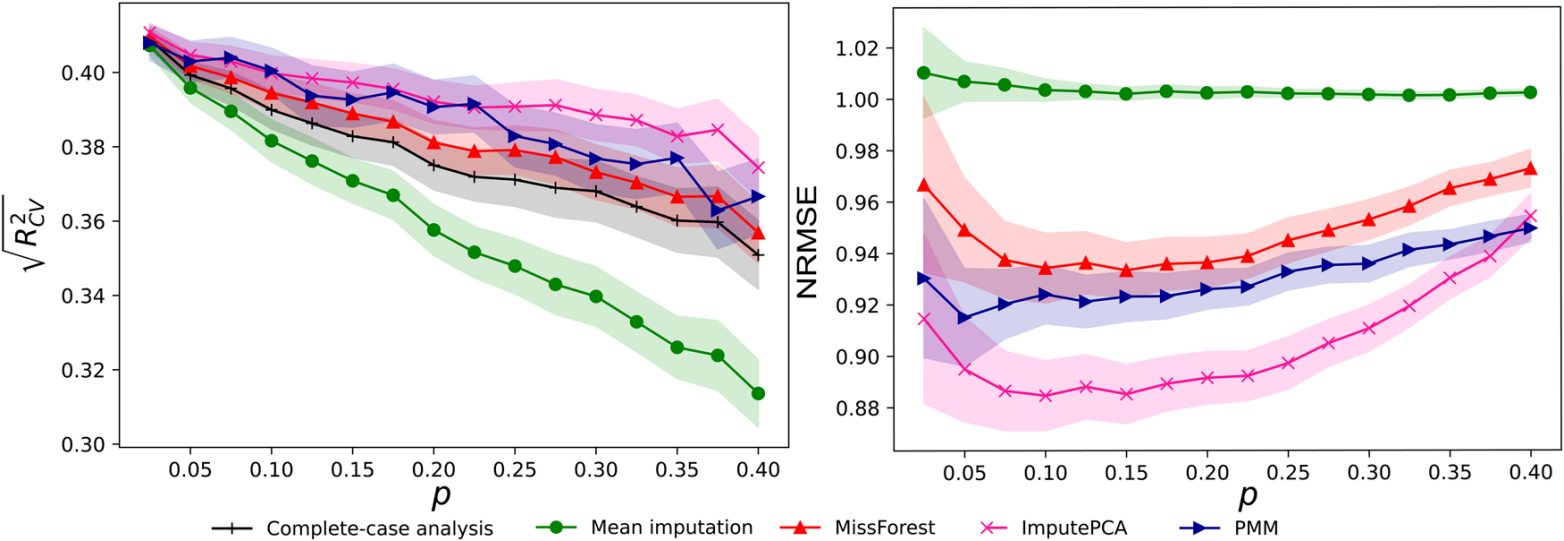
Prediction performance of models built with participants of simulated missing phenotypic measures. Prediction performance $\left(\sqrt{R_{CV}^{2}}\right)$ of models using different missing data handling strategies as a function of missingness rates *p*. Imputation accuracy (NRMSE) as a function of missingness rates *p*. The shadow areas were 95% confidence intervals.

Using the IQ data from the HCP, we conducted a simulation analysis to investigate the impact of missingness rates on prediction performance. We observed a decrease in prediction performance for all models as the missingness rate increased (Fig. 5). However, three imputation methods, namely MissForest, ImputePCA, and PMM, outperformed the complete-case analysis data, while mean imputation harmed prediction performance. Notably, ImputePCA consistently achieved the highest imputation accuracy across all missingness rates, followed by PMM and MissForest, whereas mean imputation had the lowest imputation accuracy (Fig. 5). Unlike missing connectomes, we observed that methods with higher imputation accuracy achieved better prediction performance simultaneously for missing cognitive measures.

### 3.5 Imputing missing connectomes and missing phenotypic data to rescue the maximum amount of data

**Fig. 6.**
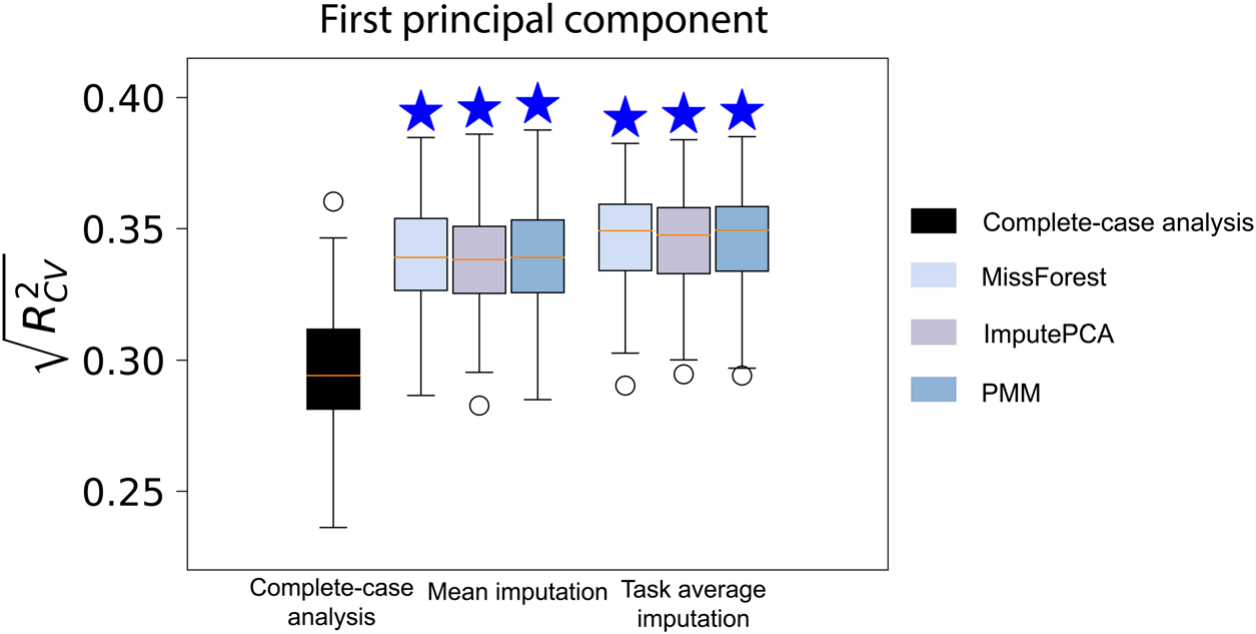
Prediction performance of models built with HBN participants with both missing connectomes and missing phenotypic measures. Missing connectomes were imputed using mean imputation and task average imputation. Missing cognitive measures were imputed using MissForest, ImputePCA, and PMM. In this example, we predicted the first principal component of 10 cognitive measures from the HBN dataset.

Finally, we present a real-world example of the benefits of data imputation in predicting cognition in the HBN dataset. In this example, we predicted the first principal component of 10 cognitive measures. The prediction performance in the complete case was 0.295 ± 0.024. Six hundred twenty-eight participants were rescued by imputing both the missing cognitive measures and connectomes. For imputing missing connectome data, we employed two simple methods: mean imputation and task average imputation. However, due to its inferior performance, meaning imputation was not used for imputing missing cognitive measures. The prediction performance was significantly greater when including the imputed data in the training set (Fig. 6). The combination of task average imputation and MissForest achieved the highest prediction performance (0.347 ± 0.018). Overall, correlation between observed and predicted was ~20% greater and explained variance was ~45% greater after including imputed data in the training set.

## 4 Discussion

In this study, we investigated rescuing missing connectomes and behavioral measures in connectome-based predictive modeling using data imputation. Imputed data were used to increase the size of the training data. For missing connectomes, we evaluated the effects of data imputation for predicting sex, age, fluid intelligence, and executive function on three independent data sets with missing connectomes. Results show that imputing missing data improved prediction performance over complete-case analysis. Moreover, simulation analyses showed that imputation strategies that best recover missing data do not necessarily signify better prediction performance, and the missing data mechanism affects the performance of different imputation methods differently. For missing phenotypic measures, improved performance over complete-case analysis was also achieved by most imputation methods. Contrary to missing connectomes, imputation strategies with higher accuracy achieved better model performance. In a real-world example, we show that including imputed connectomes and cognitive data to increase training sample sizes can significantly increase prediction performance for difficult-to-collect samples. Overall, our results show the potential of imputation methods to address incomplete data for predictive modeling.

### 4.1 Data imputation improves prediction performance

The contribution of our study is demonstrating that rescuing missing data with imputation significantly improves model performance. In the case of missing connectomes, most models built with incomplete data outperformed complete-case analysis. The improvement in prediction performance scales with the proportion of data rescued. The prediction performance in the CNP and HBN datasets showed a more significant increase than in the PNC and HCP, as the rescued data are relatively larger than the complete data. However, the gains of including more participants will be saturated when the prediction performance reaches a certain level. Notably, simple imputation methods such as task average imputation achieved the highest prediction performance in real missing data analysis. However, when connectomes are deleted with a probability proportional to motion, task average imputation fails to work. This result is because participants with high motion tend to lose more connectomes, which hinders the efficacy of imputation with the average across tasks.

Our results have implications for connectome-based predictive modeling work integrating multi-source information from distinct fMRI tasks. We showed that even leveraging simple imputation strategies can significantly improve prediction performance. A possible explanation for this result is that the intrinsic network structure, which is task-independent, contributes the most predictive information (Cole et al., 2014; Fox et al., 2005; Geerligs et al., 2015; Mill et al., 2017; Vincent et al., 2007). Moreover, recent years have witnessed the increasing availability of large-scale open data cohorts such as the UK Biobank (Sudlow et al., 2015) and the Adolescent Brain Cognitive Development (ABCD) (Karcher & Barch, 2021), which collected multimodal MRI data from tens of thousands of participants. In this context, applying imputation strategies can facilitate the use of neuroimaging data to the greatest extent possible and further accelerate the pace of establishing robust and generalizable prediction models.

Robust matrix completion, which represents the original high-dimensional data using low-rank approximation, ranks highest among all tested methods in PNC and second in HCP and CNP for predicting fluid intelligence. This result lends support to the existence of a low-dimensional space across cognitive tasks (intrinsic brain information) that is representative of the general information encoded in distinct fMRI tasks but highly informative in characterizing individual differences in cognition and behavior (Breakspear, 2017; Fox & Raichle, 2007; Gao et al., 2021; Krienen et al., 2014; Mennes et al., 2013; Shine et al., 2019).

All methods except mean imputation, which diminishes individual differences, outperformed complete-case analysis in imputing missing behavioral measures. The auxiliary variables used in the experiments are all cognitive ability measures that are correlated with each other (Dubois et al., 2018). The effectiveness of using multiple cognitive measures accords with the hypothesis that a general factor of intelligence could be derived from several cognitive tasks through factor analysis (de la Fuente et al., 2021). It is also worth noting some similar observations in meta-matching methods (He et al., 2022), translating predictive models from large-scale datasets to new unseen phenotypes in small-scale studies. In meta-matching, prediction improvements were driven by correlations between training and test meta-set phenotypes. In our case, the imputation accuracy of the target variable—in other words, how well auxiliary variables could predict the target variable—was highly correlated with prediction improvements. The similarity is not surprising given that both methods aim to include participants with phenotypes correlated with the target phenotype for model training.

### 4.2 Higher imputation accuracy does not guarantee higher prediction performance

Imputation accuracy, the accuracy of estimating missing data, was generally considered the gold standard for evaluating imputation methods (Lin & Tsai, 2020). However, our results indicate that imputation strategies that can effectively estimate missing data do not necessarily signify better prediction performance. Interestingly, our missing connectome simulation achieved the highest prediction accuracy using the average connectome across tasks. Nevertheless, this imputation strategy had the worst performance in recovering connectome data. These findings gain support from other studies investigating the relationship between imputation accuracy and prediction performance (Perez-Lebel et al., 2022). They can be partially attributed to the fact that the information recovered by imputation methods is not the most informative for prediction. In the case of connectomes with low signal-to-noise ratios (Vizioli et al., 2021), the methods with high imputation accuracy might recover noise that harms the modeling building. However, in the case of missing behavioral values (missing y), higher imputation accuracy corresponds to higher prediction performance because noisy labels severely degrade the generalization performance of machine-learning models, especially when the sample size is small (Song et al., 2022).

### 4.3 Limitations and future directions

Several potential limitations should be acknowledged when interpreting the current findings. First, we could not draw any clear conclusion about which imputation strategy is best for recovering incomplete data for prediction. Indeed, given the complexities of the problem, each method may only work for some conditions. However, our study provides preliminary insight that even a simple imputation strategy can significantly improve prediction performance and open up opportunities for future studies to integrate other imputation methods into connectome-based prediction. Second, the impact of missingness mechanisms was not considered in this pipeline. In neuroimaging studies, data are usually not missing completely at random. The imputation process could also lead to bias toward a certain subgroup of participants (Groenwold & Dekkers, 2020; Jakobsen et al., 2017). For example, head motion can sometimes predict certain phenotypes (Zeng et al., 2014), in which case the missingness indicator provides predictive modeling information (Donders et al., 2006). A crucial next step for research will be examining the potential impact of missing data mechanisms and imputation methods on datasets with larger sample sizes like the UK Biobank and ABCD. Third, the current study was performed in a functional connectome with a high-dimension and multicollinear nature. It remains unclear to what extent the imputation strategies work for other imaging modalities like structural MRI and DTI. In the next step of our research, imputation methods for other imaging modalities will be developed and examined. Fourth, in neuroimaging studies, missing phenotypic measures are less common since the cost of labeling data is much lower than that of acquiring imaging data. The missingness could be caused by combining data from different studies. Typically, cases with imputed y contain no information about the regression of y on X (Von Hippel, 2007). Imputation is only worthwhile when there are auxiliary variables available. Finally, our study primarily addresses missing data at the connectome level. Given the time series nature inherent in fMRI data, there exists a compelling opportunity to explore time series imputation techniques. This approach could be invaluable when parts of the brain exhibit lesions or when high motion artifacts occur at specific timepoints within the scan, potentially enabling the recovery of additional fMRI data. In our future research, we plan to investigate the feasibility and effectiveness of employing advanced time series imputation methods such as GRU-D (Che et al., 2016) and BRITS (Cao et al., 2018). These techniques will be leveraged to impute missing values and concurrently develop a predictive model within an integrated framework, advancing our capabilities in handling missing fMRI data with greater accuracy and precision.

## 5 Conclusion

In summary, we proposed a framework for CPM that rescues missing values in the imaging data and behavioral measures with imputation techniques. The proposed method enables us to harness the information of samples with missing data and achieve higher prediction performance compared to complete-case analysis. Moreover, we aim to raise awareness of the missing data problem in the neuroimaging community. Overall, our results suggest that participants with incomplete data are valuable for predictive modeling in neuroimaging studies, which are usually limited by the sample size.

## Supplementary Material

Supplementary Material

## Data Availability

Data were provided in part by the Human Connectome Project (HCP) (https://db.humanconnectome.org), WU-Minn Consortium (Principal Investigators: David Van Essen and Kamil Ugurbil; 1U54MH091657) funded by the 16 NIH Institutes and Centers that support the NIH Blueprint for Neuroscience Research; and by the McDonnell Center for Systems Neuroscience at Washington University. The second part of the data used in this study was supported by the Consortium for Neuropsychiatric Phenomics (https://openneuro.org/datasets/ds000030/versions/00016) (NIH Roadmap for Medical Research grants UL1-DE019580, RL1MH083268, RL1MH083269, RL1DA024853, RL1MH083270, RL1LM009833, PL1MH083271, and PL1NS062410). The third part of the data used in this study was obtained from the Child Mind Institute Biobank, the Healthy Brain Network (HBN) resource (http://www.healthybrainnetwork.org). Support for the collection of the data for the Philadelphia Neurodevelopmental Cohort (PNC) (https://www.ncbi.nlm.nih.gov/projects/gap/cgi-bin/study.cgi?study_id=phs000607.v3.p2) was provided by grant RC2MH089983, awarded to Raquel Gur, and RC2MH089924, awarded to Hakon Hakonarson. Participants were recruited and genotyped through the Center for Applied Genomics (CAG) at The Children's Hospital in Philadelphia (CHOP). Phenotypic data collection occurred at the CAG/CHOP and at the Brain Behavior Laboratory, University of Pennsylvania. Imputation methods are available from R or scikit-learn. The CPM code can be found at https://github.com/YaleMRRC/CPM.
