# Supplementary material for: Rescuing missing data in connectome-based predictive modeling
Source: Imaging Neurosci (Camb). 2024 Feb 2;2:imag-2-00071. doi: 10.1162/imag_a_00071 (PMC12224408; doi:10.1162/imag_a_00071)
Supplement: Supplementary Material [file imag_a_00071-supp.pdf]

Supplementary materials

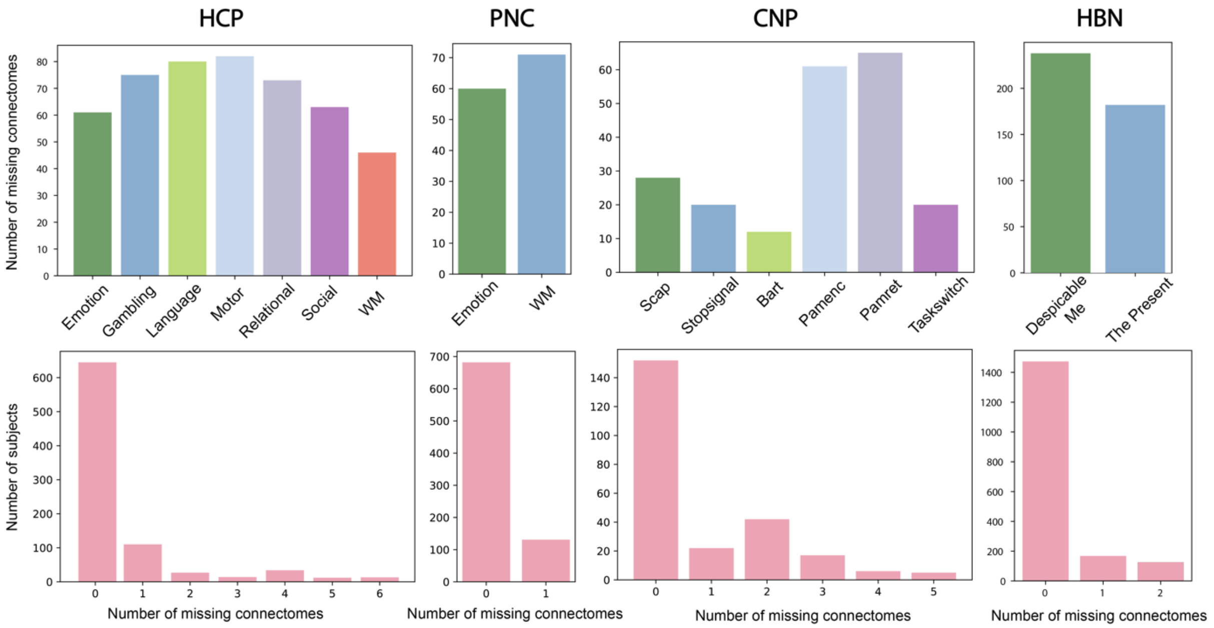

Fig. S1. Missingness of connectomes in the experimental datasets (HCP, PNC, CNP, HBN). The first row shows the number of participants that had missing connectome data for each task. The second row summarizes the counts of participants by the number of missing connectomes.

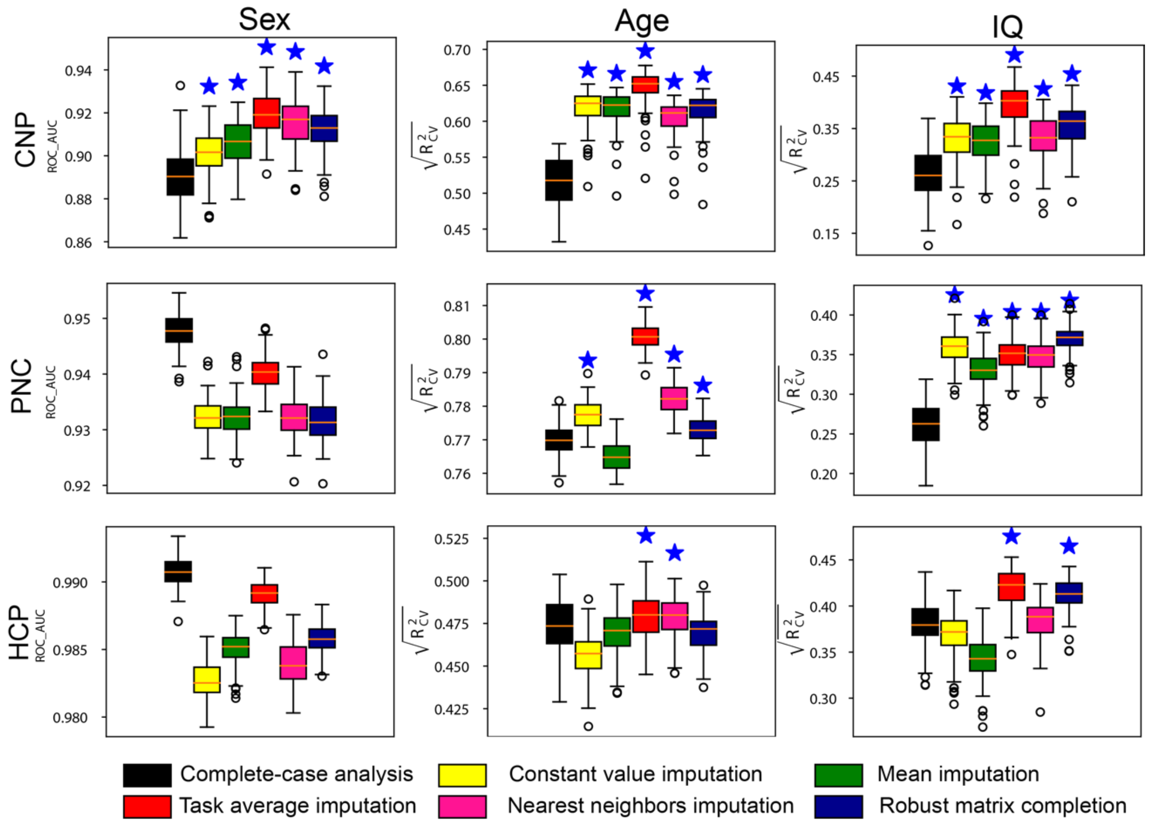

Fig. S2. Prediction performance of models built on datasets with missing connectome data. The testing set includes participants with missing connectome data. Prediction performance of sex, age and fluid intelligence based on data imputed using multiple imputation strategies in three datasets, including CNP, PNC and HCP. Stars above the boxplots indicate a significantly higher prediction performance relative to complete-case analysis ($p < 0.001$).

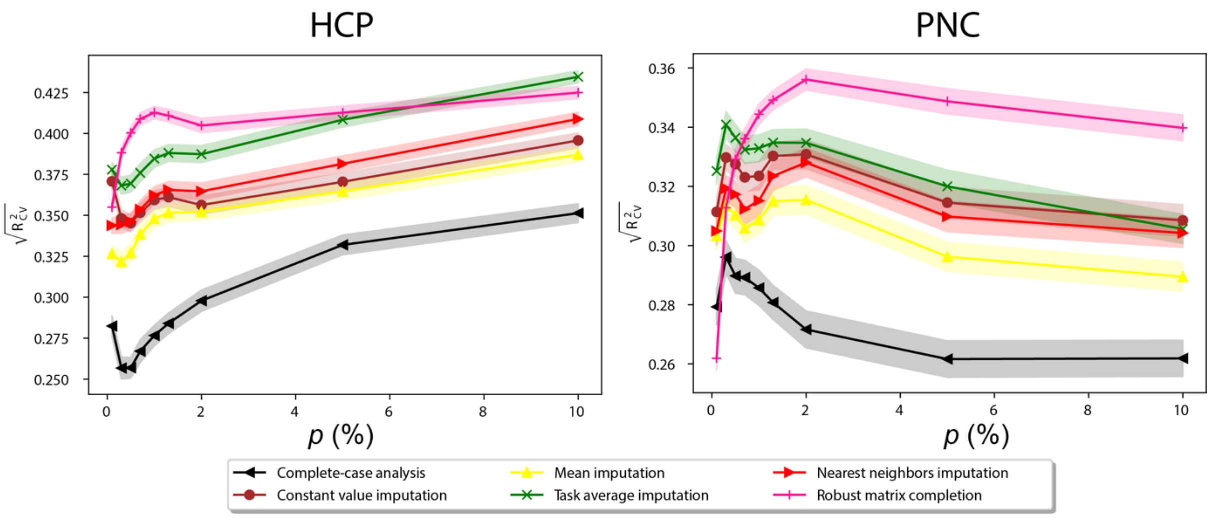

Fig. S3. Prediction performance of IQ models built with participants of missing connectomes given different feature selection percentages. These two figures correspond to IQ prediction tasks in Fig. S2, where varying percentages of features were selected. Notably, the performance of imputation methods appears to stabilize once a sufficient number of features have been selected.

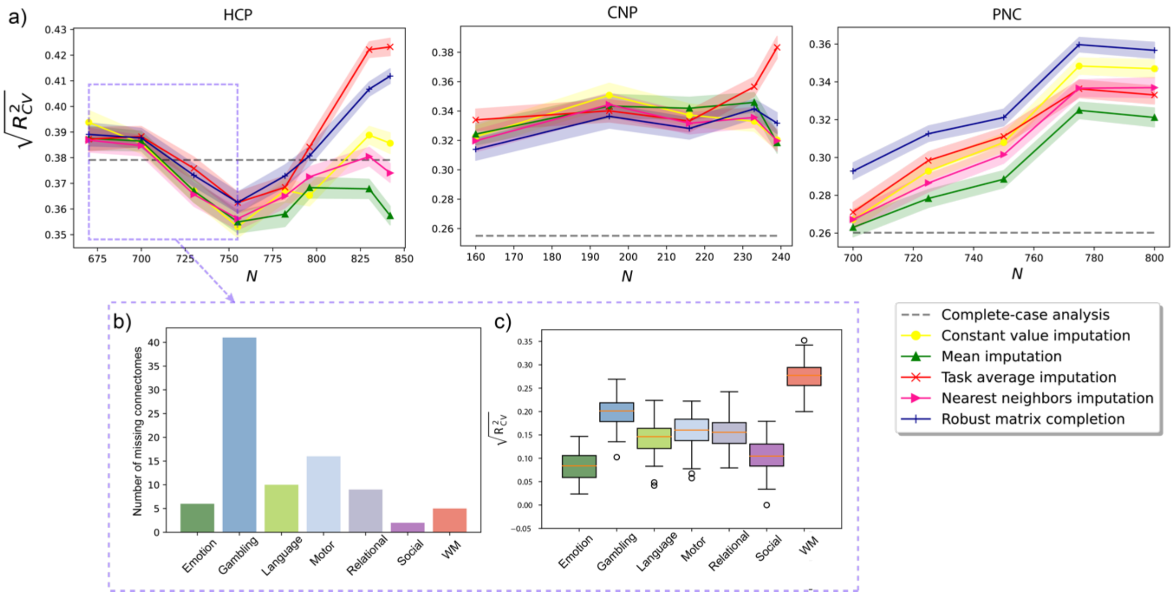

Fig. S4. Effect of including more participants with higher missingness on prediction performance (validated using all data). We iteratively added participants with higher missing connectome rates to investigate the impact of including participants with different missingness rates. a) The plots show the prediction performance as the function

of the number of participants. Participants with a higher rate of missing connectomes were incrementally added to the complete-case data. The shaded areas were 95% confidence intervals. To investigate the reason for the initial prediction performance decline in HCP dataset, we showed: b) The missingness pattern for participants who missed one connectome. The bars show the counts of missing connectomes of each task. c) Prediction performance for fluid intelligence using connectome data from each single fMRI task in HCP shown in boxplots. The model was built using the complete-case data.

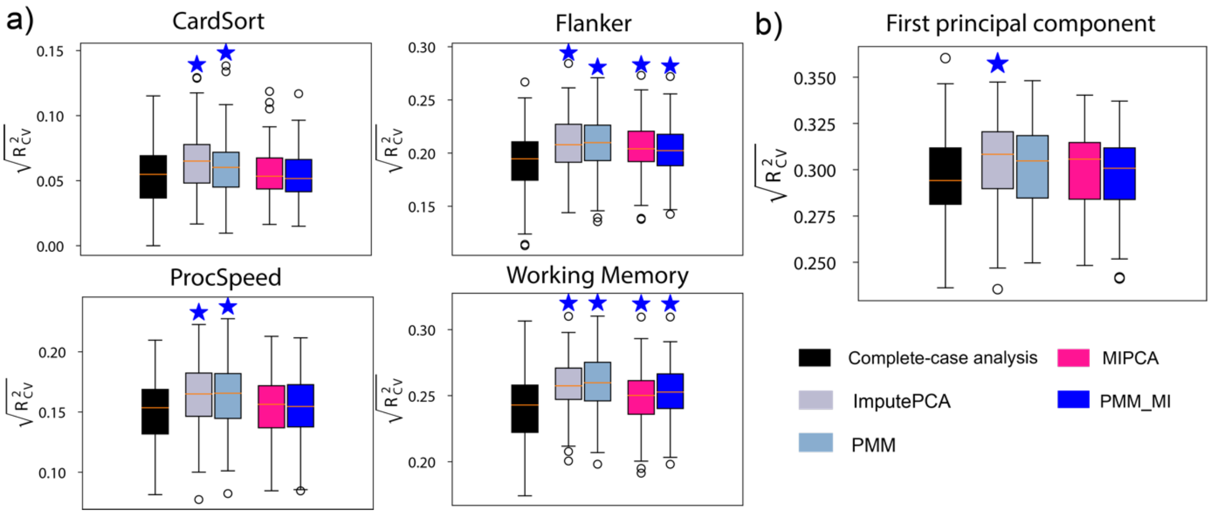

Fig. S5. Prediction performance of models built with HBN participants with missing phenotypic measures. For MICPA and PMM_MI, the performance of CPM on 10 imputed datasets was aggregated. a) Performance of predicting a single cognitive measure. b) Performance of predicting the first principal component of ten cognitive measures.
